# Providing Biological Plausibility for Exposure–Health Relationships for the Mycotoxins Deoxynivalenol (DON) and Fumonisin B1 (FB1) in Humans Using the AOP Framework

**DOI:** 10.3390/toxins14040279

**Published:** 2022-04-13

**Authors:** Annick D. van den Brand, Lola Bajard, Inger-Lise Steffensen, Anne Lise Brantsæter, Hubert A. A. M. Dirven, Jochem Louisse, Ad Peijnenburg, Sophie Ndaw, Alberto Mantovani, Barbara De Santis, Marcel J. B. Mengelers

**Affiliations:** 1 Institute for Public Health and the Environment (RIVM), 3720 BA Bilthoven, The Netherlands; marcel.mengelers@rivm.nl; 2 RECETOX, Faculty of Science, Masaryk University, Kotlarska 2, 611 37 Brno, Czech Republic; lola.bajard@recetox.muni.cz; 3 Norwegian Institute of Public Health (NIPH), 0213 Oslo, Norway; inger-lisekarin.steffensen@fhi.no (I.-L.S.); annelise.brantsaeter@fhi.no (A.L.B.); hubert.dirven@fhi.no (H.A.A.M.D.); 4 Wageningen Food Safety Research (WFSR), 6708 WB Wageningen, The Netherlands; jochem.louisse@wur.nl (J.L.); ad.peijnenburg@wur.nl (A.P.); 5 Institut National de Recherche et de Sécurité (INRS), 54500 Vandoeuvre-Lés-Nancy, France; sophie.ndaw@inrs.fr; 6 Istituto Superiore di Sanità (ISS), 00161 Rome, Italy; alberto.mantovani@iss.it (A.M.); barbara.desantis@iss.it (B.D.S.)

**Keywords:** AOP: adverse outcome pathway, HBM: human biomonitoring, HBM4EU, human biomonitoring for Europe, DON: deoxynivalenol, FB1: fumonisin B1, mycotoxins

## Abstract

Humans are chronically exposed to the mycotoxins deoxynivalenol (DON) and fumonisin B1 (FB1), as indicated by their widespread presence in foods and occasional exposure in the workplace. This exposure is confirmed by human biomonitoring (HBM) studies on (metabolites of) these mycotoxins in human matrices. We evaluated the exposure–health relationship of the mycotoxins in humans by reviewing the available literature. Since human studies did not allow the identification of unequivocal chronic health effects upon exposure to DON and FB1, the adverse outcome pathway (AOP) framework was used to structure additional mechanistic evidence from in vitro and animal studies on the identified adverse effects. In addition to a preliminary AOP for DON resulting in the adverse outcome (AO) ‘reduced body weight gain’, we developed a more elaborated AOP for FB1, from the molecular initiating event (MIE) ‘inhibition of ceramide synthases’ leading to the AO ‘neural tube defects’. The mechanistic evidence from AOPs can be used to support the limited evidence from human studies, to focus FB1- and DON-related research in humans to identify related early biomarkers of effect. In order to establish additional human exposure–health relationships in the future, recommendations are given to maximize the information that can be obtained from HBM.

## 1. Introduction

Mycotoxins are secondary metabolites that are produced by fungi upon contaminating agricultural crops in the field, or food commodities after harvest or storage. Various *Fusarium* spp. can produce deoxynivalenol (DON) and fumonisin B1 (FB1) (among other mycotoxins) and are generally associated with crop contamination in the field, such as maize and grains [1,2]. Human exposure to mycotoxins therefore mainly occurs through the consumption of contaminated food [3,4] or in an occupational setting (e.g., milling or feed processing plants [5,6]). Mycotoxin contamination is a global issue and the levels of mycotoxins in crops and derived products are monitored in many countries (for example, see [7,8,9,10,11,12,13]). Due to climate changes, it is expected that levels of mycotoxins in crops across Europe might increase due to higher temperatures and more humid conditions, and consequently the exposure of citizens to mycotoxins is likely to increase [14].

Two mycotoxins, namely DON and FB1, were placed on the 2nd priority list of substances in the EU funded project ‘Human Biomonitoring for Europe’ (HBM4EU) because of their widespread occurrence and concerns related to their possible adverse health effects in humans [15]. Short-term (acute) exposure to high levels of DON can result in gastrointestinal effects in humans reported for several outbreaks in China, with typical symptoms as vomiting, abdominal pain and diarrhoea [4,16]. Similar symptoms have been reported in studies with several animal species [4,17,18]. The European Food Safety Authority (EFSA) derived an acute reference dose (ARfD) for DON (and its derivatives/modified forms) of 8 µg/kg bw per eating occasion based on these available human data. No such value was derived for FB1, since no acute effects upon FB1 exposure have been reported, neither in human studies nor in animal studies [19].

Regarding the long-term (chronic) exposure to these mycotoxins, causal connections between exposure and adverse effects in humans have been challenging to demonstrate due to lack of established biomarkers of exposure and effect, the coexistence of several mycotoxins in the same food sources, and the inherent uncertainty in estimating dietary exposure. Therefore, animal data have been used by EFSA for the derivation of health-based guidance values (HBGVs) related to chronic exposure (i.e., tolerable daily intakes, TDIs). EFSA concluded that, in laboratory animals, the critical effect of DON was reduced body weight gain and the critical effect of FB1 was increased incidence of megalocytic hepatocytes [4,19,20,21]. The derived TDIs for DON and FB1 were both set to 1 µg/kg bw per day. In the EFSA scientific opinions on these mycotoxins, however, knowledge gaps on both hazards and exposure are expressed. No occupational exposure limits have been derived for these mycotoxins. 

The TDIs for the mycotoxins were established by EFSA based on animal studies. EFSA applied human data to set a tolerable weekly intake (TWI) for four perfluoroalkyl substances (PFAS), cadmium and dioxins [22,23,24]. The use of human data for the hazard assessment has the advantage of eliminating the uncertainty from extrapolation from animals to humans, and provides an opportunity to study vulnerable population groups. A disadvantage of the use of human data is that there is uncertainty whether observed exposure-effect relationships are causally linked, and thus, whether the studied chemical is (solely) responsible for the observed health effect. This is because no controlled exposure-effect studies can be performed, humans are exposed to a large number of chemicals simultaneously and many human studies have a cross-sectional design. As the use of human data is becoming more common, data on the mechanism of action (MOA) of chemicals is important to provide information on the causal links between exposure and health effect. Therefore, we aim to use the adverse outcome pathway (AOP) framework as mechanistic support for establishing exposure–health effect relationships for DON and FB1 in humans. 

AOPs describe, in a consistent and controlled way, a chain of consecutive key events (KEs) leading from an initial molecular perturbation (the molecular initiating event, MIE) to an adverse health effect (the adverse outcome, AO). The sequential KEs are causally related (“A leads to B”) through KE relationships (KERs). As they focus on the pathogenesis of the AO, AOPs are assumed to be “chemical independent”: an AOP can be relevant to every chemical whose mechanism fits into the appropriate MIE/KE(s). Nonetheless, AOPs constitute a very useful tool in chemical risk assessment by providing a conceptual framework to collect, structure, and evaluate the supporting evidence for the biology underlying the mechanisms of toxicity [25]. Especially for substances for which limited human-relevant toxicological data are available, like most mycotoxins, AOPs can be used to gain mechanistic evidence of biological plausibility and causality to the identified epidemiological associations between mycotoxin exposure and suggested adverse human health effects.

In this study, we describe the dietary exposure estimates and adverse human health effects that are reported for DON and FB1 in the general population and in occupational settings. A literature search was performed to identify studies that reported human health effects as a result of the exposure to these mycotoxins. As solid evidence for human exposure–health relationships is lacking, we further show that the AOP framework can be used to structure evidence from in vitro and animal studies, thereby supporting this relationship by providing information on the mechanisms underlying reported human health effects of mycotoxins. Suggestions to establish future exposure health relationships are provided. 

## 2. Results

### 2.1. Exposure Estimates

#### 2.1.1. Dietary Exposure Estimates in the General Population

The exposure of the general population to mycotoxins predominantly occurs through food consumption. Several different *Fusarium* fungi can contaminate crops in the field and produce DON and FB1, or other forms of these mycotoxins, such as acetyl-derivatives of DON and FB2-4, which are structurally similar to DON and FB1, respectively [4,19]. Plants can also metabolize the mycotoxins into glucoside-metabolites, like DON-3-glucoside. These derivatives and metabolites can be converted back to their parent compound in the gut, thereby adding to the exposure of the original mycotoxin [26,27]. The dietary exposure to DON and FB1 in European citizens was assessed by EFSA in 2017 and 2014, respectively (Table 1) [3,4]. The sources driving the exposure to DON and FB1 are grains and grain-based products, and bread other than maize-based (DON) and maize and pasta (FB1) [3,4].

The difference between the lower and the upper bound exposure scenarios was much larger for the FB1 dietary exposure assessment than for that of DON, in both the infants, children and adults. Chronic exposure to FB1 is probably not an issue of concern in all European countries, in contrast to DON, considering these large differences in exposure estimates. This difference likely results from a large contribution of occurrence data analysed below the detection limits, and reflects the greater uncertainty regarding the dietary intake of FB1 and its modified forms. Nonetheless, the highest exposure estimates for some European countries in the 95th percentile exceed the derived TDIs for both DON and FB1 in some population groups. Moreover, exposure to DON is expected to increase in the general population as a result of climate change [14]. The same trend is likely regarding FB1 [28].

#### 2.1.2. Exposure Estimates in the Occupational Setting

Occupational exposure may occur in workplaces due to mycotoxins contained in organic matters such as feed, food, or waste. Several studies have reported the prevalence of mycotoxins in dust samples in grain elevators, suggesting an occupational exposure through inhalation and skin contact, and a few studies addressed occupational exposure to DON by assessing internal or external exposure, as further described below.

DON was quantified in settled dust collected from grain elevators [29,30,31], and bakeries [6,32]. Concentrations of DON in settled dust varied from 10 ng/g to up to 1 µg/g. The concentration of DON was determined in aerosols generated during grain handling [33]. Grain workers were shown to be frequently exposed to DON. The highest level of exposure was about 60 ng/m^3^. Dust samples from personal airborne samplers were also analysed by Ndaw et al. (2021) to assess external exposure of grain elevator workers to mycotoxins. DON was quantified in 54% of the air samples [34]. The median concentration was 6.0 ng/m^3^ and the highest level of exposure was 80.1 ng/m^3^. In these studies where occupational co-exposure to multiple mycotoxins was assessed, DON was among the most prominent mycotoxins. However, it still remains to be determined whether the detected concentrations in settled dust and air samples cause internal exposure after intake through inhalation or skin, and could give rise to health impairments. Knowledge regarding the toxicokinetics of mycotoxins following exposure via inhalation, through dermal contact, or hand-mouth contact in the workplace are still lacking. Information on the absorption of DON via lungs and skin in humans and associated health effects is needed to evaluate the risk.

Only five biomonitoring studies reporting urinary concentrations of DON among workers were identified. A multi-biomarker approach was used to assess occupational exposure to mycotoxins in different settings, including grain mills, a bread dough company, swine production, and in grain elevators [5,6,34,35,36]. Mycotoxin biomarker levels were determined in urine samples from workers. To discern between the occupational exposure and the background dietary exposure, control groups without expected occupational exposure were also enrolled in three of the five studies. The comparison of results from workers and from controls makes it possible to take into account the dietary exposure and to have a better understanding of the role of occupational contributions in the total burden of mycotoxins. Several mycotoxins were detected in urine samples and, similar to the external exposure results, DON was the most prominent mycotoxin. An overview of these studies is given in Supplementary Data A, Table S1. We appraised the quality of these studies as moderate, following the LaKind scoring criteria (also summarized in Supplementary Data A) [37]. The main reasons for the moderate quality appraisal were the limited number of workers included [34,35,36], the limit of quantification higher than the current state of the art [5,6], and a sampling strategy that included a single spot urine sample [5,6,36] collected at random time [6,36].

Additional occupational exposure of mill workers is apparently low as concluded by Follman et al., in contrast to Viegas et al., who concluded that workplace exposure adds significantly to the exposure resulting from ingestion of mycotoxin-contaminated food among workers from bread dough companies and swine production farms [5,6,36]. Ndaw et al. also came to the conclusion of higher DON exposure for grain elevator workers when compared to previously published data on non-occupational exposure [34]. 

There is very little information on the occupational exposure to fumonisins. This is probably because there is no practical human exposure biomarker, as most fumonisin is excreted via the faeces and only a low percentage of fumonisin is excreted via urine upon oral exposure [38]. Occupational exposure to multiple mycotoxins was assessed in a swine production and a fresh bread dough company [5,6]. FB1 urinary levels were below the limit of detection for controls and workers in both studies. Exposure to FB1 was also studied among grain elevator workers during wheat and maize harvest [34]. While quantified in 72% of airborne samples, FB1 was not detected in urinary samples.

### 2.2. Health Effects

#### 2.2.1. Deoxynivalenol

To assess whether human health effects have been described for DON, the most recent EFSA Scientific Opinion was consulted [4]. EFSA concluded that the evidence of adverse health effects in humans due to chronic exposure to DON is lacking as there were no published studies that described this. Our literature search (performed at the end of 2019, the end of 2020, and repeated in December 2021) did not reveal any new human studies on DON, except for a cross-sectional study [39] which found higher mycotoxin concentrations in blood and urine in children with autism than in controls, and significant correlations between mycotoxins, including FB1, and clinical manifestations and comorbidities in children with autism. Due to the cross-sectional design of the study, it is impossible to conclude whether mycotoxin exposure was a cause of clinical manifestation of autism or whether autism comorbidities (e.g., gastrointestinal dysfunction and leaky gut, differences in gut microbiota, differences in IgG and cytokines) may explain the higher concentrations of mycotoxins in autistic children. Consequently, this study was not applicable to add supporting evidence of causal adverse effects of mycotoxin exposure on autism. DON has the potential to impair protein synthesis and cell respiration, which is a plausible mechanism linking DON to reduced body weight in laboratory rodents [4,21]. Due to the lack of adequate human studies, it is still uncertain whether impaired protein synthesis and/or body weight decrease are relevant chronic health effects in humans.

Although no chronic human health effects have been identified for exposure to DON, human relevant effects of acute exposure to DON are evident, as indicated by the ARfD that was derived from human data by EFSA [4]. The health effects of acute DON outbreaks (incidental high exposure) as described by EFSA were nausea, vomiting, diarrhea, abdominal pain, headaches, dizziness, fever, and in severe cases, bloody stool. No lethality was reported.

#### 2.2.2. Fumonisin B1

The most recent EFSA Scientific Opinion was consulted to identify studies on the chronic effects of FB1 to humans [19]. The document reports that several clinical effects have been mentioned in humans, such as oesophageal cancer, liver cancer, neural tube defects (NTD) or growth impairment, but so far none of these have been causally related to fumonisin exposure. In the Opinion, human studies that link FB1 exposure to liver toxicity/liver cancer or NTD were described, and also provided information of reported effects in animal studies on these endpoints. Only two relevant human studies were found, one for liver toxicity/cancer and one for NTD, which both describe a human health effect and included an estimation of FB exposure in humans. Persson et al. did not find a statistically significant association between FB1 exposure and liver cancer [40]. The authors conducted case-control studies nested within two large prospectively designed cohorts in China: the Haimen City Cohort and the General Population Study of the Nutritional Intervention Trials cohort in Linxian. In the Haimen City Cohort, nail FB1 levels were determined in 271 hepatocellular cancer (HCC) cases and 280 controls. In the General Population Nutritional Intervention Trial, nail FB1 levels were determined in 72 HCC cases and 147 controls. In each population, odds ratios (OR) and 95% confidence intervals (95%CI) from logistic regression models estimated the association between measurable FB1 and HCC, adjusting for hepatitis B virus infection and other factors. A meta-analysis that included both populations was also conducted. The analyses revealed no statistically significant association between FB1 in nails and HCC in either Haimen City (OR = 1.10, 95%CI = 0.64–1.89) or in Linxian (OR = 1.47, 95%CI = 0.70–3.07). Similarly, the pooled meta-analysis showed no statistically significant association between FB1 exposure and HCC (OR = 1.22, 95%CI = 0.79–1.89). Although FB1 has been demonstrated to cause liver tumors in animal models [41,42], the authors concluded that there was no statistically significant association between FB1 exposure and HCC in these two Chinese cohorts. Using the Effective Public Health Practice Project (EPHPP) appraisal form [43], we appraised the quality of the study by Persson et al. as moderate (Supplementary Data B), which was in line with appraisal by others [44].

Regarding the relationship between FB1 and NTD, one population-based case-control study by Missmer et al. concluded that the findings suggested that fumonisin exposure increased the risk of NTD, proportionate to dose, up to a threshold level, at which point fetal death may be more likely to occur [45]. The authors examined whether maternal exposure to fumonisins increased the risk of NTDs in the offspring. Fumonisin exposure was estimated from a postpartum sphinganine (Sa)/sphingosine (So) (Sa/So) ratio, the suggested biomarker for fumonisin exposure measured in maternal serum, and from maternal recall of periconceptional corn tortilla intake. After adjusting for confounders, moderate (301–400) compared with low (≤100) consumption of tortillas during the first trimester was associated with increased ORs of having a NTD-affected pregnancy (OR = 2.4; 95% confidence interval, 1.1–5.3). No increased risks were observed at intakes higher than 400 tortillas (OR = 0.8 for 401–800, OR = 1.0 for >800). Based on the postpartum Sa/So ratio, increasing levels of fumonisin exposure were associated with increasing ORs for NTD occurrence, except for the highest exposure category (Sa/So > 0.35). No difference between the associations was found when serum folate concentrations were included in the analysis. We appraised the quality of this publication also as moderate because the intake of FB1 was indirectly estimated via recalled tortilla intake and the suggested exposure biomarker Sa/So ratio (Supplementary Data C). 

In addition, Marasas et al. noticed the presence of studies that reported a high FB1 occurrence (in maize) in certain areas, e.g., a specific province in China, South Africa and Guatemala, which also appear to be areas with high frequencies of NTD in newborns, as reported in other studies [46,47,48,49,50]. This favors the hypothesis of an association between FB1 exposure and NTD prevalence. This is, however, circumstantial evidence of a link between FB1 exposure and NTD. Nevertheless, multiple animal studies have been identified that show that FB1 dose-dependently induced NTD in the offspring of mice or whole (rat and mice) embryos cultured ex vivo [51,52,53,54,55,56,57,58]. The prevalence (and severity) of the NTDs differed between the studies, which is likely due to differences in genetic background of the strains and timing of exposure. Yet, EFSA concluded that the evidence for fumonisin-induced NTD from animal studies was overall inconclusive, despite some indications in mice [19]. This conclusion may be too cautious; indeed, the NTD induced by FB1 in rodents may hint to gene-environment interactions. On the other hand, no dose-response relationship can be derived from the available data, in order to evaluate any possible relationship between human exposure and teratogenicity in humans.

Altogether, our search did not identify evidence in humans for a health effect associated with chronic DON exposure. Only the acute effect of DON exposure, vomiting, is well-established in humans. In case of FB1, one epidemiological study, with some circumstantial evidence, and several animal studies support a link between FB1 exposure and NTD. However, EFSA concluded that causality could not be demonstrated in humans (EFSA, 2018). Therefore, the mechanistic association between the molecular effects of exposure to DON or FB1, and the health effects in humans was further explored using the AOP framework.

### 2.3. Adverse Outcome Pathways

When using the limited available human evidence, supporting mechanistic data like AOPs, are very important to support an exposure-health outcome relationship. The AOP framework was here used as a tool to provide mechanistic evidence for the suggested exposure-health outcome relationships considering the mechanisms of toxicity induced by DON and FB1 in humans. 

#### 2.3.1. Deoxynivalenol

For DON, emesis was identified as an acute effect in both humans as well as pigs and mink [4,17]. Reduced body weight gain in experimental animals was identified as the critical chronic effect for human risk assessment. For DON, we drafted a putative AOP, with two main routes, based mostly on the information provided in the most recent EFSA Scientific Opinion on DON [4]. Briefly, DON-binding to ribosomes (MIE) can subsequently activate mitogen-activated protein kinases (MAPK) (KE1). Activation of MAPKs results in a variety of different effects, which may explain the various effects observed upon DON exposure. Two main routes that further mediate the DON-induced anorexia, emesis, and subsequent growth suppression were identified. Following the ‘pro-inflammatory route’, MAPK activation leads to an increase in pro-inflammatory cytokines (KE2), which induces an inflammatory response (KE3), including, for example, the activation of nuclear factor kB (NF-kB), target of rapamycin (TOR), or cyclo-oxygenase-2 (COX-2). Intestinal tissue damage (KE4), as a consequence of the inflammatory response (KE3), would lead to the DON-induced AO. The second route leading to the AO involves an increase in the secretion of gut satiety hormones (KE5) as a response to MAPK activation (KE1). These hormones can activate receptors in the abdominal vagus afferent, that communicate with the areas in the brain involved in food/feed uptake [4].

Solid support for associations between DON and almost all currently proposed KEs was identified in the literature (for example, [59,60,61,62,63,64]). This substantiates the conclusion that the proposed AOP would constitute a mechanism for DON-induced adverse effects like emesis and subsequent weight loss in animals and, possibly, in humans. However, more evidence would be required to further validate the AOP, starting with evaluating the confidence in KERs and essentiality of the KEs. 

There were no chronic human studies identified that demonstrate that the critical effect observed in laboratory animals, reduced body weight gain, is relevant to humans. The AOP was therefore not developed further, as the frame of this research focuses on exposure–health outcome relationships that have been identified in humans. 

#### 2.3.2. Fumonisin B1

**Figure 1:**
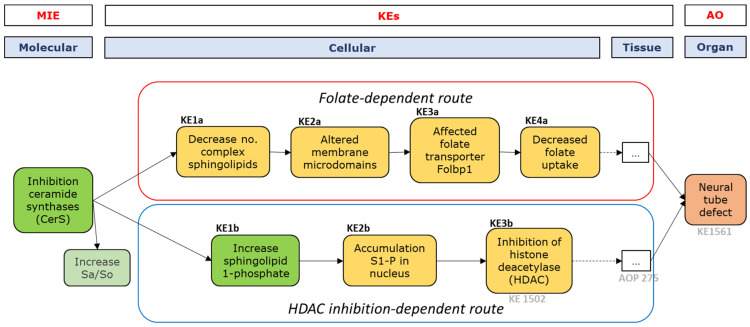
Putative AOP for CerS inhibition-mediated NTD. Boxes in green: sufficient evidence available in support of KEs; boxes in orange/yellow: limited evidence available in support of KEs. The increase of Sa/So is proposed as a biomarker of effect, expected to result from the MIE, but is not a KE per se. AOPs and KEs already present in AOPWiki have been indicated in grey.

For FB1, the mechanisms underlying the association between FB1 exposure and NTD was also drafted in a putative AOP. We elaborated on this AOP in greater detail, as compared to the putative AOP for DON, and proceeded to a more in-depth evaluation of the studies and KEs involved in the AOP. The drafted AOP describes two possible chains of events leading from the inhibition of ceramide synthases (CerS), the suggested MIE to NTD, to the AO: a folate-dependent route and a histone deacetylase (HDAC) inhibition-dependent route (see Figure 1). 

It is well documented that the last KEs in both routes (i.e., decreased folate uptake and inhibition of HDAC) are involved in the development of NTD [65,66,67,68,69], but how CerS inhibition results in decreased folate uptake and/or inhibition of HDAC has not been extensively described. Therefore, the focus of this study was to obtain more insight into the KEs and KERs, starting from CerS inhibition up to decreased folate uptake and inhibition of HDAC, and to collect available evidence for this AOP.

##### MIE and First Key Events—Sphingolipid Metabolism

**Figure 2:**
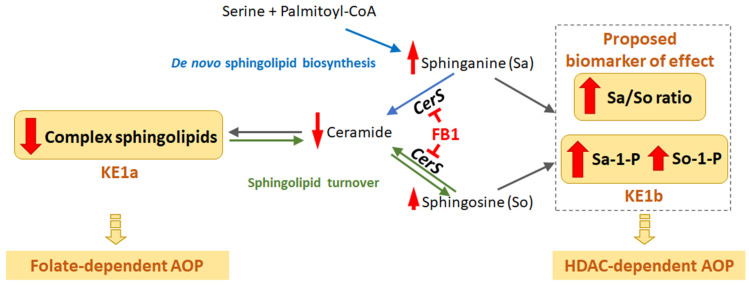
Overview of the effects of FB1 on sphingolipid metabolism with links to the proposed AOPs and biomarkers of effect (adapted from EFSA, 2018). Red arrows indicate the consequences of FB1-induced inhibition of ceramide synthases (CerS).

The proposed MIE in this putative AOP is the inhibition of CerS, which is a key enzyme in sphingolipid metabolism. Ceramide synthases are enzymes that catalyze for one the acylation of Sa to form (dihydro-)ceramide and more complex sphingolipids [19,70], and also the reacylation of So that is derived from the turnover of complex sphingolipids (Figure 2) [19,71]. Fumonisins are regarded as structural analogues of Sa and So [72,73], and FB1 is an inhibitor of all six ceramide synthase isoforms [74,75]. This inhibition results in an increase of Sa, So, and, often, Sa/So ratio in the presence of FBs. FB1-induced inhibition of ceramide synthases results in, among others, two possible effects: a decrease in the level of ceramides and complex sphingolipids [72,76] and an increase in the phosphorylated forms of Sa and So [52]. The first effect is proposed as a KE (KE1a) for the folate-dependent route, while the second effect is proposed as a KE (KE1b) for the HDAC inhibition-dependent route. 

An increase in Sa/So ratio, as reported in various animal and some human studies [77,78], is also expected from the inhibition of CerS, as Sa can still be formed by de novo sphingolipid biosynthesis, whereas So formation from ceramide is expected to decrease in time, also due to a decrease in complex sphingolipids (Figure 2).

##### Folate-Dependent Route, KE-1-4

One possible route leading to NTD involves a decrease in folate uptake, which has been reported to induce NTDs [68,79,80]. FB1 exposure was shown to affect folate transport and decrease folate uptake in Caco-2 cells [81], and to reduce folate levels in mouse embryonic and placental tissues [53]. Furthermore, folate administration attenuated FB1-induced NTDs [53], supporting a critical role of folate in FB1-induced NTDs. It was shown that depletion in complex sphingolipids (some of these are gangliosides) can impact folate uptake and ganglioside administration partially rescues the FB1-induced decrease in folate levels in embryonic tissues [53]. However, the detailed molecular and cellular processes linking the effects on complex sphingolipids to the effects on folate are more speculative. Folate transporter Folbp1 is a glycosylphosphatidylinositol (GPI)-anchored protein, and sphingolipids were shown to be involved in endocytic trafficking of GPI-anchored proteins [82]. Marasas and colleagues proposed that a depletion of sphingolipids (KE1a), caused by the inhibition of CerS (MIE) could alter membrane microdomains enriched in cholesterol and sphingolipids, also called lipid rafts (KE2a), thereby affecting the folate transporter Folbp1 trafficking (KE3a) and folate amounts available in maternal blood, as well as in embryonic tissues [53,83].

CerS inhibition primarily results in accumulation of sphingoid bases and a decrease in levels of ceramides, which are the precursors of complex sphingolipids (for review, see [84,85]. It is therefore highly plausible that the inhibition of ceramide synthase would result in a decrease in levels of complex sphingolipids (KER1a), and it is supported by several studies, mostly in vitro and using FB1 as an inhibitor of CerS [81,86,87]. However, it remains to be determined whether there are differences between different complex sphingolipids (e.g., sphingomyelin, gangliosides, etc.) and whether some are more impacted and/or more essential for the downstream events than others.

The hypothesis that a relationship exists between altered complex sphingolipids (KE1a) and lipid rafts (KE2a, expressed as KER2a) is highly plausible since several of these sphingolipids, such as gangliosides, are enriched in lipid rafts. It is also supported by some in vitro studies [88,89]. The hypothesis that alteration of membrane microdomains affects localization, stability, and/or function of GPI-anchored proteins (such as folate transporter Folbp1) (KER3a) is also biologically plausible and supported by some in vitro circumstantial evidence. For instance, alteration of membrane composition correlates with the endocytic trafficking of the folate receptor [90,91] and it appears to be well accepted that GPI-anchors tend to target proteins to lipid rafts [92]. However, the existence of lipid rafts is still a topic of debate in the scientific community, mostly due to technical difficulties in characterizing and manipulating them. For the same reasons, in vivo empirical evidence for KER2a and KER3a and for the essentiality of KE2a is lacking and might be challenging to provide.

##### HDAC Inhibition-Dependent Route, KE1-3

Another possible route leading to NTD involves the inhibition of HDACs, since HDAC inhibitors, such as valproic acid and trichostatin A, have been reported to induce NTD [66,93]. A putative AOP linking HDAC inhibition to NTD was developed as part of another EU project [94], with proposed KEs following from HDAC inhibition as MIE being ‘imbalance of histone acetylation’ and resulting ‘altered gene expression’ at the molecular level, leading to ‘altered differentiation’ at the tissue level resulting in ‘NTD’ as AO. In the following, we summarize available empirical evidence, directly or indirectly supporting the proposed KEs/KERs between CerS inhibition and HDAC inhibition. The proposed KEs/KERs can then, when combined with the AOP linking HDAC inhibition to NTD, form the basis for the development of an AOP linking CerS inhibition to NTD.

Gardner et al. performed in vitro studies showing that FB1 causes increased acetylation of histones and decreased HDAC activity, pointing to an FB1-induced inhibition of HDACs (KE3b) [95]. They exposed mouse embryonic fibroblasts (MEFs) from LM/Bc mice to FB1 and observed increased acetylation of lysine residues in H2 and H3 (H2BK12, H3K9, H3K23) and a decreased HDAC activity in the nuclei of FB1-exposed MEFs compared to controls. As described above, (FB1-induced) inhibition of CerS can result in an increase in Sa and So and their phosphorylated forms (Sa-1-P and So-1-P), especially Sa and Sa-1-P. Indeed, Gardner et al. observed an increase in Sa, So, Sa-1-P, and So-1-P in FB1-exposed MEFs, both in the cytosol and in the nucleus. 

We propose the event ‘Increase S-1-P’ as first KE (KE1b), followed by ‘Accumulation of S-1-P in nucleus’ as second KE (KE2b), linking to the final KE ‘HDAC inhibition’ (KE3b) in the HDAC-inhibition-dependent route. In a functional study with MCF-7 cells, Hait et al. showed that So-1-P specifically binds to HDAC1 and HDAC2, and inhibits their enzymatic activity, indicating that HDACs are direct intracellular targets of So-1-P (KER between KE2b and KE3b) [96]. These HDACs play major roles in neural development processes and So-1-P is one of the few known endogenous HDAC inhibitors [97]. The study of Hait et al. focussed on the role of So-1-P as epigenetic regulator of gene expression, and no information on the possible role of Sa-1-P as HDAC inhibitor was reported. Gardner et al. propose Sa-1-P to be the critical factor causing HDAC inhibition in their study with MEFs, as they found the amount of nuclear Sa-1-P to be 30-fold higher than the amount of nuclear So-1-P in FB1-exposed MEFs. As we do not have the data to decide on whether either So-1-P or Sa-1-P is the most critical in HDAC inhibition resulting from CerS inhibition, we used ‘accumulation of S-1-P in the nucleus’ (including both So-1-P and Sa-1-P) as KE2b in the putative AOP. It is further of interest to note that So-1-P is also able to activate So-1-P receptors, a class of G protein-coupled receptors [98], but whether this So-1-P receptor activation leads to HDAC inhibition has not yet been described.

Sa-1-P and So-1-P are formed from Sa and So, respectively, in the cytosol, mainly by sphingosine kinase 1 (Sphk1), and in the nucleus, by Sphk2 [99]. Mizugishi et al. showed that sphingosine kinase-null mice exhibited a deficiency of So-1-P that severely disturbed neurogenesis, including neural tube closure, showing the critical role of these enzymes in neural development [100]. In the study of Gardner et al., pre-treatment of MEFs with a selective Sphk1-inhibitor or a selective Sphk2-inhibitor caused a decrease in nuclear Sa-1-P levels. Even though effects were more pronounced after treatment with the Sphk2-inhibitor, it cannot be concluded whether S-1-P-mediated HDAC inhibition upon CerS inhibition is (mainly) due to Sa/So phosphorylation in the nucleus or whether phosphorylation in the cytosol also plays a (critical) role.

To conclude, the increase of S-1-P in the cell (KE1b) upon CerS inhibition (KER1b) is well established. The subsequent accumulation of S-1-P in the nucleus (KE2b) resulting from the increased S-1-P levels (KER2b) is biologically highly plausible. The inhibition of HDAC (KE3b) resulting from the S-1-P accumulation in the nucleus (KER3b) has been studied in vitro providing empirical, supportive evidence. Although the KEs and KERs of this putative AOP can be considered as biologically highly plausible, only few studies are available to provide the supportive evidence. Most available studies to support this putative AOP are in vitro studies. Also, the studies showing FB1-induced S-1-P accumulation in the nucleus and HDAC inhibition (KE2b and KE3b) were performed using mouse embryonic fibroblasts, and the studies on S-1-P-induced HDAC inhibition (KER3b) were performed in human breast cancer cells (MCF7). Evidence obtained in cells/tissues that play a role in neural tube development in humans would increase the confidence in the relevance of this proposed AOP.

### 2.4. Establishing (Future) Exposure–Health Relationships

In order to establish exposure–health relationships in the future, human biomonitoring (HBM) strategies should be optimized to obtain as much information as possible from the human data. Different approaches are sometimes necessary, depending on the route of exposure. In the general population, mycotoxin exposure frequently occurs through diet, whereas in an occupational setting, inhalation and dermal exposure to mycotoxins may also add to the total exposure of the workers.

The chronic exposure to DON and FB1 in Europe is, for most subpopulations, around or slightly above their TDI. In both cases, overall assessment (uncertainty) factors (for intra- and inter-species variability) of 100 have been applied to different points of departure. Therefore, it is uncertain whether current chronic exposure levels will lead to measurable adverse effects in the general population, in particular when focusing on apical endpoints related to critical effects in animals (i.e., in mice). The putative AOPs that have been described above relate to adverse outcomes in humans. Nevertheless, it is anticipated that future exposure–health relationships in humans for DON or FB1 should not only focus on their (ultimate) adverse outcomes but also on effect biomarkers related to earlier KEs. For DON as well as FB1, several branches have been described in the putative AOPs. It is still too early to translate these KEs into relevant effect biomarkers but it is likely that such KEs may lead to preclinical effects in humans, especially in those cases where the dietary exposure is relatively high and on a daily basis. With respect to the selection of effect biomarkers, the toxicokinetics of these mycotoxins should also be taken into account since we touch upon the toxicodynamics when searching for effect biomarkers. It has been shown that DON has a relatively short elimination half-life in adults, approximately 3–4 h [101,102]. This means that its half-life is shorter than the average exposure interval (during daily meals containing cereals products), which indicates that DON does not accumulate in the human body, and internal exposure levels (e.g., in blood) will show a large variation within the day. However, it is not certain whether fluctuating internal doses will be mirrored by fluctuating concentrations of effect biomarkers. There is little known on the toxicokinetics of FB1 in humans but it seems that it is not eliminated as rapidly as DON and it has been suggested that enterohepatic circulation could occur, for example in rats and swine [103,104]. Nevertheless, it has been shown that FB1 may alter concentrations of certain sphingolipids or sphingolipid complexes. As is shown in the proposed putative AOP of FB1, these are rather early KEs and it remains to be seen if a relatively low, chronic exposure to FB1 can be causally related to changes in the effect biomarkers concentrations. Therefore, it should be kept in mind that exposure–health relationships in humans are best detected in highly exposed (sub)populations. Furthermore, prospective epidemiological studies should focus on these (sub)populations, whereas retrospective studies related to incidental high exposures could also be used to test the applicability of an effect biomarker.

## 3. Discussion

In this study, we reviewed the available data on the exposure–health relationship of the mycotoxins DON and FB1. Since human studies did not allow the identification of unequivocal chronic health effects after exposure to DON and FB1, the AOP framework was used to collect, structure and evaluate mechanistic evidence that underlie the adverse effects found in the identified human studies. Moreover, the proposed KEs can contribute to the identification of early biological (pre-clinical) effects that may be further explored as effect biomarkers for DON or FB1 exposure.

### 3.1. Exposure–Health Relationships

#### 3.1.1. Exposure Estimates

Where EFSA estimates the exposure to DON and fumonisins in Europe using consumption data and EU-wide monitoring data, there is limited information on the exposure estimates of DON and FB1 in non-European countries. Staple foods in Africa, Asia, and Latin-America are contaminated with mycotoxins like DON and fumonisins, in addition to aflatoxins, ochratoxin and zearalenone [12,105,106,107].

Exposure biomarkers can be used to identify the level of exposure to the mycotoxins in all populations and will provide an aggregated exposure assessment taking into account different routes of exposure, including oral, inhalation, and dermal. 

#### 3.1.2. Biomarkers

For DON, the established exposure biomarker is free DON measured in urine, after deconjugation of its glucuronidated metabolites DON3GlcA and DON15GlcA [108]. Approximately 70% of the ingested DON is excreted in urine [26,101,109]. Therefore, urine is a good matrix to analyse the exposure to DON. However, care should be taken regarding which type of urine samples are collected for biological monitoring. Spot urine samples will not be adequate for exposure assessment. The first urine samples collected in the morning can provide a better picture, but preferably 24 h pooled urine samples should be used to describe exposure adequately [102]. Unfortunately, no effect biomarkers have been identified for DON [110]. 

Concerning effect biomarkers for DON, there is a need to propose and develop biomarkers that are related to the referred health outcome, i.e., weight loss. For that purpose, mechanistic knowledge that allows for drafting an AOP can also serve to identify some possible effect biomarkers anchored on putative KEs. Omics-based methods, such as transcriptomics allow identification of a number of differentially expressed genes involved, for example, in gut inflammatory response (KE3). The value of this approach can be primarily assessed through in vitro assays using human intestinal cells exposed to low doses of DON and identifying differentially expressed genes compared to unexposed cultures. Additionally, their sensitivity and reliability must be assessed in large epidemiological studies. 

For FB1, estimating the exposure in biological matrices is less straightforward. Approximately 1–2% of FB1 is excreted in urine [38]. Several studies have, however, suggested that the increase of the Sa/So (sphingolipid) ratio in biological fluids can be used as a sensitive biomarker of fumonisin exposure and early biological effects [38,77,78]. Sa and So levels in biological fluids have not yet been investigated in occupational settings. 

Although the increased Sa/So ratio (or Sa-1-P/So-1-P) has been suggested as a biomarker of exposure and effect, the validity of the Sa/So ratio as a biomarker in humans remains uncertain [78,111]. This is partly due to the fact that Sa and So occur and vary naturally in human blood [111,112]. Furthermore, there is no human reference value for physiologically normal levels of these sphingoid bases or the Sa/So ratio. 

A biomarker of effect should provide evidence of biochemical or biological alterations before the onset of disease, thereby increasing the biological likelihood of exposure–health outcome associations. For instance, Missmer et al. reported association between Sa/So levels and OR of NTD [45], but the studies reporting associations between this biomarker and health outcomes are still limited and more evidence is needed to support its use in HBM studies on FB1 exposure. In this context, the proposed AOPs provide some mechanistic support for the Sa/So ratio as a biomarker of an early effect. However, although the increase in Sa/So ratio is a consequence of the MIE, it is not a KE of the AOP per se. 

Also, Riley et al. found a correlation between FB1 intake and changes in Sa-1-P and Sa-1-P/So-1-P ratios in blood in humans [78]. The second branch of our proposed AOP provides mechanistic support for using these ratios as early biomarkers of effect for NTD. Mechanistic evidence may also provide support for linking the biomarker to liver toxicity, as the identified MIE could also target other cells, like hepatocytes, and thereby induce other effects, like liver toxicity [19]. Although this supporting evidence was not examined systematically in the present study. 

Based on the AOP that was developed for FB1, other effects biomarkers may be proposed. As FB1 is recognized as an inhibitor of HDAC, a central regulator of gene expression, a more open chromatin structure resulting in deregulated gene expression is expected following exposure to this mycotoxin. Therefore, in vitro assays that target chromatin structure or downstream genes expression can be used to start the development of novel effect biomarkers.

### 3.2. Adverse Outcome Pathways

After examining the MIE, KEs and KERs leading from inhibition of CerS to NTD, it appears that there is accumulating evidence for a relationship between FB1 exposure and NTD in the foetus. This information can be used to focus FB1-related research regarding this adverse effect in humans. However, as a next step, more in-depth evaluation of the evidence supporting the essentiality of each KE and the biological plausibility of the KERs needs to be performed for both routes of the AOP. Also, it must be noted that liver toxicity, indicated as FB1’s critical effect in animals, may still be relevant in humans even though human data that link this effect to FB1 exposure are lacking.

#### 3.2.1. Applicability

Since the evidence supporting the proposed AOPs for both FB1 and DON comes almost exclusively from animal and in vitro studies, it is still unclear to which extent the mechanisms are applicable to humans. In addition, it is unknown whether FB1 can cross the placental barrier in humans. The data appear to be lacking in humans, and the reports from animal studies are inconsistent, probably due to differences between species and stages of pregnancy [113,114,115,116]. However, as sphingolipids are endogenous compounds, an alteration of maternal sphingolipid (ratios) as a result of fumonisin exposure, may also affect the fetus’ sphingolipid circulation via the feto-placental vasculature [117].

Regarding FB1-induced NTDs in humans, the critical window of exposure would be the very early stages of pregnancy, when the neurulation process occurs [118]. Defining the levels of FB1 exposure that are expected to induce NTDs would require more quantitative information for the AOPs, combined with data from toxicokinetic modeling. 

#### 3.2.2. Branching of the Adverse Outcome Pathway

The proposed AOPs describe two linear chains of causally linked events leading from the initiating event (i.e., the MIE) tot the effect (i.e., AO). These biological perturbations may also lead to other downstream effects or constitute connections to other pathways. 

For instance, regarding FB1, the MIE induces a decrease in the levels of ceramide [119], which play an important role in key cellular processes, in addition to neural tube development [120,121,122]. Folate deficiency has also been associated with other adverse outcomes, including anemia, cardiovascular disease, and neurological problems [123]. 

In one route of the AOP, lipid rafts are affected as a consequence of (FB1-induced) inhibition of CerS and subsequent inhibition of complex sphingolipids. In the proposed AOP, this impacts folate uptake, which is a KE towards the NTD status. However, lipid rafts may also impact other pathways. For example, they have been proposed as signaling hubs in cancer progression [124]. These membrane microdomains may also interfere with So-1-P, suggesting some possible cross-talks between both branches of the proposed AOP [125]. 

The AOPs, or parts of, described here may also underly toxicity of other stressors. For example, potato glycoalkaloids have also been associated with NTDs in humans [126,127], but the mechanisms underlying these effects remain poorly understood. Interestingly, these stressors have been shown to alter biosynthesis of cholesterol, which is a key component of membrane microdomains [128]. In addition, no associations between glycoalkaloids and NTDs were found in mothers with higher folate consumption, consistent with the involvement of folate in the adverse pathway [127]. Some elements of our proposed AOP (from KE2 to AO) might therefore also constitute a mechanism underlying NTDs induced by potato glycoalkaloids.

*Alternaria* toxins from *A. alternata* f. sp. *lycopersici* (AAL-toxins) are also structural analogues of sphinganine. In addition, like FB1, AAL-toxins can inhibit ceramide synthase [129]. This gives rise to the notion that, in addition to FB1, AAL-toxins may also contribute to NTD in humans after (oral) exposure. However, toxicological data are largely lacking for these mycotoxins. As studies increasingly report the levels of various *Alternaria* toxins in food and feed [9,130,131], attention can be given to the combined exposure to FB1 and AAL-toxins from food products, where possible. 

### 3.3. Human Biomonitoring

In order to establish exposure–health relationships in the future, biomonitoring strategies can be used to optimize the data that can be obtained from human biomonitoring. The ideal study design for a biomonitoring study of DON in the general population should include collection of urine samples over a 24 h period (preferably two independent 24 h periods to increase the representativeness of the sample) and pooling of urine samples. Very sensitive analytical procedures are needed to measure the unconjugated or conjugated forms of DON. Alternatively, enzymatic hydrolysis could be performed to convert the conjugated forms to free forms, but special care should be taken to ensure that the enzymatic hydrolysis procedures used have a high activity and specificity to form the free metabolites [132]. Ideally, the food products consumed during the study period are collected by using a duplicate diet study design for analysis of the presence of mycotoxins in the consumed diets [133]. It is considered important to repeat the biomonitoring studies using the same subjects covering different seasons and different years in order to study seasonal and annual trends in the exposure of DON. Annual trends are especially important since climate changes are expected to contribute to higher levels of DON and other mycotoxins in the diet. 

For HBM of FB1 exposure, urine samples are not considered an appropriate matrix since only a very small fraction of FB1 metabolites are excreted in urine. Use of faecal materials and/or blood should be considered to improve the biomonitoring of FB1. In addition, hair and nail were suggested as matrices for long-term FB1 exposure in laboratory animals, including monkeys, as well as humans [40,134,135]. Although it may prove difficult to relate concentrations of FB1 in hair to reliable exposure estimates, it may be used to find associations between FB1 exposure and health effects. 

In occupational settings, biomonitoring studies should be combined with the assessment of the external exposure through inhalation and dermal contact to ascertain these exposure pathways. Environmental monitoring, including personal air sampling and hand wipes, should be implemented throughout the work shift. The study population should be representative of the scenario of exposure of the workplace and include a control group to take into account the dietary exposure. The control group can be employees of the same workplace without known occupational exposure to mycotoxins or adults from the general population and from the same geographical area. The sampling strategy for urine samples is of great importance to recognize what the workplace environment might be, adding to the exposure already occurring because of food intake. The limited knowledge regarding DON’s toxicokinetics via different exposure routes makes it difficult to define the ideal sampling time. The collection of urine samples over a 24 h period can be challenging to implement at work and at home. Ndaw et al. suggested that collecting multiple samples over several days, with a standardized schedule including pre-shift, post-shift, and first morning urine samples, may help assess the workplace’s contribution to the total exposure [35]. 

Furthermore, although effect biomarkers of mycotoxins have been rarely used in human biomonitoring studies, they can assist in the risk assessment of single mycotoxins and, more importantly, of their mixtures by bridging combined exposure to health outcomes. 

### 3.4. Recommendations

Critical data gaps still exist regarding the potential health effects of DON and FB1 in humans. Although the assessment of human exposure through urinary concentration and dietary assessment indicates that several populations and population groups are highly exposed to these mycotoxins, linking exposure to adverse health effects in human studies has not yet been possible. To bridge the data gaps identified in this study, we recommend the following (in random order and not reflecting rank of importance). 

Comprehensive studies should be performed on co-occurrence of mycotoxins and their modified forms in grains and food products for human consumption [4]. Special attention can be paid to the (development of appropriate methods of) analysis of *Alternaria* (AAL-)toxins, since these are structurally similar to FB1. 

Further research could focus on the exposure and effect biomarkers of FB1 in humans (e.g., Sa/So ratio), and the determinants of the internal exposure (e.g., factors contributing to rate of degradation and excretion and interplay with other substances in food and gut microbiota).

Also, more research could be dedicated to interindividual differences and the role of concurrent genetic and non-genetic (e.g., nutritional, age) risk factors for NTD, in combination with FB1 exposure (which is difficult to study since NTD are rare malformations).

Epidemiological studies on the health effects of the mycotoxins may include a higher number of participants (both male and female), with good exposure estimates (external and/or internal doses). Also, more research can be dedicated to the (uptake) kinetics of DON in humans via the non-dietary exposure routes, such as inhalation.

It is necessary to standardize criteria for biomonitoring sampling and analysis for these mycotoxins (e.g., suitable sample type (pooled 24h urine) and timing, analytical targets (biomarkers) and analytical methods, including quantification and detection limit threshold values).

In addition, more in vivo/ex vivo research could be dedicated to assess the applicability of the FB1 AOP to humans, especially regarding the ability of FB1 to cross the placental barrier. Studies focused on providing information on the essentiality of KEs in the proposed AOPs would help increase the level of confidence in the AOP. 

## 4. Conclusions

Human studies did not allow the identification of unequivocal chronic health effects after exposure to DON and FB1. Therefore, the AOP framework was used to structure additional mechanistic evidence from in vitro and animal studies on the identified adverse outcomes of DON and FB1. The supporting mechanistic evidence from the AOPs can be used to support the limited evidence on exposure–health relationships from human studies and to focus DON- and FB1-related research in humans regarding the adverse outcome for DON (reduced body weight gain) and FB1 (NTD). In order to establish additional human exposure–health relationships in the future, recommendations are given to bridge the data gaps identified in this study, for example to focus research on validating exposure and effect biomarkers and identifying the determinant of internal exposure. 

## 5. Materials and Methods

This study is a product of the collaboration within HBM4EU, work package 13 on ‘Establishing exposure–health relationships’. Two mycotoxins, DON and FB1, were placed on the second list of HBM4EU’s priority substances because of their widespread occurrence and concerns related to their possible adverse health effects in humans, and, as such, were the mycotoxins under consideration for this study [15]. To establish the exposure–health relationships for these mycotoxins in humans, a first search was dedicated to human cohort studies that included prolonged exposure to DON and FB1. As these cohort studies are lacking, other literature was consulted to obtain an insight into the human-relevant adverse effects of these mycotoxins. As little evidence was found, supporting evidence was sought using the AOP framework as a tool to collect and evaluate the supporting evidence for the biology underlying the mechanisms of toxicity.

### 5.1. Literature Searches

EMBASE was used as a database to identify newly published information on DON and FB1 after the most recent EFSA Scientific Opinions from 2017 and 2018 [4,19]. The search strings were developed after consultation with an information specialist at RIVM. See Supplementary Data D for the search string for DON. This search was performed by the end of 2019, 2020, and repeated by the end of 2021. All studies were exported separately and imported together in an Endnote database. The studies were manually screened by one individual for relevance based on title and abstract.

To identify a first set of studies to aid the development of the putative AOP, not only studies investigating FB1, but also other chemicals interfering with sphingolipid metabolism (e.g., myriocin, FTY720 (fingolimod), AAL toxin TA, and australifungins) were considered. Studies related to the MIE, AO, KE, and KERs of the putative AOP for FB1 were initially identified using the EMBASE database. Several search strings were created and used to collate the literature (see Supplementary Data E). All studies were exported separately and imported together in an Endnote database. Title and abstract were screened by two individuals for possible relevant articles regarding the MIE and KE1 (and KE2). The following exclusion criteria were used: studies on Arabidopsis and Candida (i.e., in vivo data on all species other than mammals), conference abstracts, and book chapters.

### 5.2. Appraisal of Studies

The appraisals of the pivotal human studies on the chronic effects of the mycotoxins were conducted using the EPHPP appraisal forms [43] by three individuals independently and discussed until agreement was reached. The appraisal of the occupational biomonitoring studies was conducted according to the LaKind scoring [37] by one individual. 

The evaluation of the literature relating to the putative AOP, Kes, and KERs was conducted by expert opinion. Studies were grouped in tables and relevant information was summarized. Based on the gathered evidence, the proposed KEs in the putative AOPs leading from the MIE to the AO were identified. The identified relevant information on the KEs and KERS are summarised in the text.

## Figures and Tables

**Table 1:** Chronic daily dietary exposure estimates (µg/kg bw/day) for DON and FB1 and respective TDIs as reported by EFSA in 2018, 2017, and 2014.

	Total DON ^1^ [4] TDI ^3^ 1 µg/kg bw/day [4]		Total Fumonisins ^2^ [3] TDI 1 µg/kg bw/day [19]	
	Mean (LB-UB ^4^)	P95 ^5^ (LB-UB)	Mean (LB-UB)	P95 (LB-UB)
Infants and children	0.2–2.0	0.7–3.7	0.04–1.8	0.2–4.1
Adults	0.3–0.7	0.5–1.4	0.05–0.6	0.09–1.3

^1^ Total DON: including 3-AC-DON, 15-Ac-DON and DON-3-glucoside; ^2^ Total fumonisins: fumonisin B1, B2 and B3; ^3^ TDI: tolerable daily intake; ^4^ LB-UB: Lower bound-Upper bound; ^5^ P95: dietary exposure in the 95th percentile of the distribution, i.e., high exposure.

## Data Availability

Not applicable.

## Supplementary Materials

The following supporting information can be downloaded at: https://www.mdpi.com/article/10.3390/toxins14040279/s1. Supplementary Data A: Overview and appraisal of occupational HBM studies; Table S1: Occupational HBM studies on DON exposure and LaKind appraisal score; Supplementary Data B: Appraisal Persson et al. (2012); Supplementary Data C: Appraisal Missmer et al. (2006); Supplementary Data D: EMBASE search string for DON; Supplementary Data E: EMBASE search string for FB1.
