# Supplementary material for: Providing Biological Plausibility for Exposure–Health Relationships for the Mycotoxins Deoxynivalenol (DON) and Fumonisin B1 (FB1) in Humans Using the AOP Framework
Source: Toxins (Basel). 2022 Apr 13;14(4):279. doi: 10.3390/toxins14040279 (PMC9030459; doi:10.3390/toxins14040279)
Supplement: Supplementary file 1 [file toxins-14-00279-s001.zip › toxins-1658206-supplementary.pdf]

toxins

MDPI

Article

Providing Biological Plausibility for Exposure–Health Relationships for the Mycotoxins Deoxynivalenol (DON) and Fumonisin B1 (FB1) in Humans Using the AOP Framework

Annick D. van den Brand Lola Bajard, Inger-Lise Steffensen, Anne Lise Brantsæter, Hubert A.A.M. Dirven, Jochem Louisse, Ad Peijnenburg, Sophie Ndaw, Alberto Mantovani, Barbara De Santis and Marcel J.B. Mengelers

Supplementary data

A – Overview and appraisal of occupational HBM studies

Table S1. Occupational HBM studies on DON exposure and LaKind appraisal score

Study	Occupational setting/number of workers and controls	Analytical method/Biomarkers			Results			
		**Substances studied**	**Biomarker of exposure /Matrix/ Sampling time**	**Method LOD – LOQ** (µg/L)	Creatinine-adjusted /non–adjusted urinary DON concentration (µg/g creatinine/µg/L) [a]	**Main conclusions on exposure**	**Additional information**	**LaKind scoring**[a]
Follmann et al., 2016 (Germany)	Mill workers (n=17, male n=12) Control group (n=13), employees from another company	DON Citrinin Ochratoxin A Zearalenone	**DON, DOM-1**[1] OTA, OTα CIT, DH-CIT[2] ZEN, αZEL, βZEL[3] /urine/ random during work shift	LC-MS/MS LOD/LOQ DON 0.15/0.3 LOD/LOQ DOM-1 0.1/0.2	**DON** - 100 % positive samples Male workers Median (µg/L): 5.4 (range 1.27 – 113.8) Median (µg/g): 4.8 (range 1.5 – 12.0) Controls Median (µg/L): 6.8 (range 1.01 – 14.6) Median (µg/g): 5.7 (range 1.1 – 13.4) **DOM-1** Male workers – 54 % positive samples Median (µg/L): 0.114 (range LOD – 0.216) Median (µg/g): 0.07 (range LOD – 0.3) Controls	No significant differences for DON between male workers and controls Range of DOM-1 similar in the two cohorts	Other mycotoxins detected Workers, >LOD OTA, 100% OTα, 33% CIT, 100% DH-CIT, 100% ZEN, 100% αZEL, 33% βZEL 17% Controls, >LOD OTA, 77% OTα, 62% CIT, 100% DH-CIT, 100% ZEN, 100% αZEL, 46% βZEL 23%	14

					Median (µg/L):<br>0.05 (range LOD – 0.18)<br>Median (µg/g):<br>0.05 (range LOD – 0.17)			
Viegas et al., 2018 (Portugal)	Workers from fresh dough company (n=21)<br><br>Control group (n=19), employees from another company	DON<br>Aflatoxin B1/2, G1/2<br>Aflatoxin M1<br>Alternariol<br>Ochratoxin A<br>Citrinin<br>T-2 toxin<br>HT-2 toxin<br>Fumonisin B1/2<br>Zearalenone<br>Enniatins A/A1/B/B1<br>Beauvericin	**DON**<br>**DON-3-GlcA**<br><br>AFB1/2, AFG1/2<br>AFM1<br>Alternariol metabolites[4]<br>OTA metabolites[5]<br>CIT, DH-CIT<br>T-2<br>HT-2, HT-2-4GlcA[6]<br>FB1, FB2<br>ZEN, αZEL, βZEL[3], ZAN, ZEN-14-GlcA[7]<br>EnA/A1/B/B1<br>Beauvericin<br><br>urine/ random, during work shift between<br>11 a.m and 4 p.m	LC-MS/MS<br><br>LOD/LOQ<br>DON 0.5/2<br><br>LOD/LOQ<br>DON-3-GlcA<br>1.24/4.14	**DON-3-GlcA**<br>Workers<br>95 % samples >LOD<br>14 % samples >LOQ (n=3)<br>Range: 12.6 – 64.5 µg/g<br>Mean: 34.87 µg/g, SD 17.45 µg/g<br><br>Controls<br>58 % samples >LOD<br>0 % samples >LOQ<br><br>**DON**: not detected	DON-GlcA was the most prominent biomarkers found in both groups (based on the number of samples >LOD)<br><br>DON-GlcA was at the highest levels in samples from workers	Other mycotoxins detected<br>Workers, >LOD<br>AFM1, 14%<br>EnB, 14%<br>CIT, 29%<br>DH-CIT, 14%<br>OTA, 48%<br>2′R-OTA, 10%<br><br>Controls, >LOD<br>AFM1, 5%<br>EnB, 11%<br>CIT, 58%<br>DH-CIT, 11%<br>OTA, 68%<br>2′R-OTA, 21%	16
Viegas et al., 2019 (Portugal)	Swine production Workers (n=25)<br><br>Control group (n=19), employees from another company	DON<br>Aflatoxin M1<br>Citrinin<br>Ochratoxin A	**DON-3-GlcA**<br><br>AFM1<br>OTA<br>CIT, DH-CIT<br><br>urine/ during work shift between<br>11 a.m and 1 p.m	LC-MS/MS<br><br>LOD/LOQ<br>DON-3-GlcA<br>1.24/4.14	**DON-3-GlcA**<br>Workers<br>60 % samples >LOD<br>52 % samples >LOQ (n=13)<br>Range: 22 – 71.1 µg/L<br>Median: 32.8 µg/L<br><br>Controls<br>58 % samples >LOD<br>0 % samples >LOQ	DON-GlcA was one of the most prevalent biomarkers found in the worker group (based on the number of samples >LOD). Only workers presented quantifiable level of DON-GlcA	Mycotoxins detected<br>Workers, >LOD<br>AFM1, 16%<br>EnB, 4%<br>CIT, 8%<br>DH-CIT, 12%<br>OTA, 88%<br><br>Controls, >LOD<br>AFM1, 5%<br>EnB, 11%<br>CIT, 58%<br>DH-CIT, 11%<br>OTA, 68%<br>2′R-OTA, 21%	15
Ndaw at al., 2021a (France)	Grain elevator Workers (n=3)	DON<br>Aflatoxin B1<br>Ochratoxin A<br>T-2 toxin<br>HT-2 toxin	**DON**<br><br>AFB1<br>AFM1<br>OTA , OTα	HR-MS/MS<br><br>LOQ<br>DON 0.05	**DON**<br>100% samples >LOQ (n=9)	DON was one of the most prevalent biomarkers. The low number of	Mycotoxins detected<br>Samples >LOQ<br>AFB1, 55%	13

		Fumonisin B1<br>Zearalenone	T-2<br>HT-2<br>FB1<br>ZEN, αZEL, βZEL,<br><br>urine/ pre-shift, post-shift and first morning void samples		Range: 3.9 – 18.8 μg/L (2.75– 21.4 μg/g)<br>Median: 14.4 μg/L (12.1 μg/g)	workers include d and the lack of a control group do not enable to draw any conclusions on the magnitude of the occupational exposure.	AFM1, 44%<br>OTA, 100%<br>ZEN, 66%<br>αZEL, 22%	
Ndaw at al., 2021b (France)	Grain elevator Workers (n=18)	DON<br>Aflatoxin B1<br>Ochratoxin A<br>T-2 toxin<br>HT-2 toxin<br>Fumonisin B1<br>Zearalenone	**DON**<br><br>AFB1<br>AFM1<br>OTA , OTα<br>T-2<br>HT-2<br>FB1<br>ZEN, αZEL, βZEL,<br><br>urine/ pre-shift, post-shift and first morning void samples; 24-h urines samples and up to three days	HR-MS/MS<br><br>LOQ<br>DON 0.05	**DON**<br>98% samples >LOQ (n=195)<br>Range: <LOQ – 154 μg/L (<LOQ– 123 μg/g)<br><br>Median: 14.5 μg/L (12.5 μg/g)<br>Pre-shift median: 9.9 μg/L (8.10 μg/g)<br>Post-shift median: 22.1 μg/L (12.7 μg/g)	DON was one of the most prevalent biomarkers. Concentrations of DON were higher than previously reported concentrations from the general population. Concentrations of DON appeared to be higher in post-shift samples than in pre-shift samples.	Mycotoxins detected Samples >LOQ<br>OTA, 76%<br>ZEN, 99%<br>αZEL, 52%<br>βZEL, 33%<br>T-2, 4%<br>HT-2, 4%	12

[a] The lower the LaKind score the better the overall quality (possible range 9-27).

[1] De-epoxy DON; [2] dihydrocitrinone; [3] α- and β-zearalenol; [4] alternariol, alternariol–monomethyl ether, altenuene; [5] ochratoxin A, 2'R ochratoxin A, 10 hydroxyochratoxin A, ochratoxin α ; [6] HT-2-4-glucuronic acid ; [7] zearalenone, zearalanone, zearalenol-14-glucuronic acid.

B – Appraisal Persson et al. (2012)

QUALITY ASSESSMENT TOOL FOR QUANTITATIVE STUDIES

COMPONENT RATINGS

A) SELECTION BIAS

(Q1) Are the individuals selected to participate in the study likely to be representative of the target population?

1 Very likely
2 Somewhat likely
3 Not likely
4 Can't tell

(Q2) What percentage of selected individuals agreed to participate?

1 80 - 100% agreement
2 60–79% agreement
3 less than 60% agreement
4 Not applicable
5 Can't tell

RATE THIS SECTION	STRONG	MODERATE	WEAK
See dictionary	1	2	3

B) STUDY DESIGN

Indicate the study design

1 Randomized controlled trial
2 Controlled clinical trial
3 Cohort analytic (two group pre + post)
4 Case-control
5 Cohort (one group pre + post (before and after))
6 Interrupted time series
7 Other specify ____________________
8 Can't tell

Was the study described as randomized? If NO, go to Component C.

N Yes

If Yes, was the method of randomization described? (See dictionary)

No Yes

If Yes, was the method appropriate? (See dictionary)

No Yes

RATE THIS SECTION	STRONG	MODERATE	WEAK
**See dictionary**	1	2	3

C) CONFOUNDERS

(Q1) Were there important differences between groups prior to the intervention?

1 Yes
2 No
3 Can't tell

The following are examples of confounders:

1 Race
2 Sex
3 Marital status/family
4 Age
5 SES (income or class)
6 Education
7 Health status
8 Pre-intervention score on outcome measure

(Q2) If yes, indicate the percentage of relevant confounders that were controlled (either in the design (e.g. stratification, matching) or analysis)?

1 80 – 100% (most)
2 60 – 79% (some)
3 Less than 60% (few or none)
4 Can't Tell

RATE THIS SECTION	STRONG	MODERATE	WEAK
**See dictionary**	1	2	3

D) BLINDING

(Q1) Was (were) the outcome assessor(s) aware of the intervention or exposure status of participants?

1 Yes
2 No
3 Can't tell

(Q2) Were the study participants aware of the research question?

1 Yes
2 No
3 Can't tell

RATE THIS SECTION	STRONG	MODERATE	WEAK
**See dictionary**	1	2	3

E) DATA COLLECTION METHODS

(Q1) Were data collection tools shown to be valid?

1 Yes

2 No
3 Can't tell

(Q2) Were data collection tools shown to be reliable?
1 Yes
2 No
3 Can't tell

RATE THIS SECTION	STRONG	MODERATE	WEAK
**See dictionary**	1	2	3

F) WITHDRAWALS AND DROP-OUTS

(Q1) Were withdrawals and drop-outs reported in terms of numbers and/or reasons per group?
1 Yes
2 No
3 Can't tell
4 Not Applicable (i.e. one time surveys or interviews)

(Q2) Indicate the percentage of participants completing the study. (If the percentage differs by groups, record the lowest).
1 80 -100%
2 60 - 79%
3 less than 60%
4 Can't tell
5 Not Applicable (i.e. Retrospective case-control)

RATE THIS SECTION	STRONG	MODERATE	WEAK	
**See dictionary**	1	2	3	Not Applicable

G) INTERVENTION INTEGRITY

(Q1) What percentage of participants received the allocated intervention or exposure of interest?
1 80 -100%
2 60 - 79%
3 less than 60%
4 Can't tell

(Q2) Was the consistency of the intervention measured?
1 Yes
2 No
3 Can't tell

(Q3) Is it likely that subjects received an unintended intervention (contamination or co-intervention) that may influence the results?
4 Yes
5 No
6 Can't tell

H) ANALYSES

(Q1) Indicate the unit of allocation (circle one)
community organization/institution practice/office individu

(Q2) Indicate the unit of analysis (circle one)
community organization/institution practice/office individu

(Q3) Are the statistical methods appropriate for the study design?
1 Yes
2 No
3 Can't tell

(Q4) Is the analysis performed by intervention allocation status (i.e. intention to treat) rather than the actual intervention received?
1 Yes
2 No
3 Can't tell

GLOBAL RATING

COMPONENT RATINGS
Please transcribe the information from the gray boxes on pages 1-4 onto this page. See dictionary on how to rate this section.

**A**	**SELECTION BIAS**	**STRONG**	**MODERATE**	**WEAK**	
		1	2	3	
**B**	**STUDY DESIGN**	**STRONG**	**MODERATE**	**WEAK**	
		1	2	3	
**C**	**CONFOUNDERS**	**STRONG**	**MODERATE**	**WEAK**	
		1	2	3	
**D**	**BLINDING**	**STRONG**	**MODERATE**	**WEAK**	
		1	2	3	
**E**	**DATA COLLECTION METHOD**	**STRONG**	**MODERATE**	**WEAK**	
		1	2	3	
**F**	**WITHDRAWALS AND DROPOUTS**	**STRONG**	**MODERATE**	**WEAK**	
		1	2	3	Not Applicable

<u>GLOBAL RATING FOR THIS PAPER (circle one):</u>

1	STRONG	(no WEAK ratings)
2	MODERATE	(one WEAK rating)
3	WEAK	(two or more WEAK ratings)

With both reviewers discussing the ratings:

Is there a discrepancy between the two reviewers with respect to the component (A-F) ratings?

No Yes

If yes, indicate the reason for the discrepancy

1 Oversight
2 Differences in interpretation of criteria
3 Differences in interpretation of study

<u>**Final decision of both reviewers (circle one):**</u>

1	**STRONG**
2	**MODERATE**
3	**WEAK**

C – Appraisal Missmer et al. (2006)

QUALITY ASSESSMENT TOOL [illegible] QUANTITATIVE STUDIES

EPHPP
Effective Public Health Practice Project

COMPONENT RATINGS

A) SELECTION BIAS

(Q1) Are the individuals selected to participate in the study likely to be representative of the target population?

1 Very likely
2 Somewhat likely
3 Not likely
4 Can't tell

(Q2) What percentage of selected individuals agreed to participate?

1 80 - 100% agreement
2 60–79% agreement
3 less than 60% agreement
4 Not applicable
5 Can't tell

RATE THIS SECTION	STRONG	MODERATE	WEAK
See dictionary	1	2	3

B) STUDY DESIGN

Indicate the study design

1 Randomized controlled trial
2 Controlled clinical trial
3 Cohort analytic (two group pre + post)
4 Case-control
5 Cohort (one group pre + post (before and after))
6 Interrupted time series
7 Other specify ____________________
8 Can't tell

Was the study described as randomized? If NO, go to Component C.

N Yes

If Yes, was the method of randomization described? (See dictionary)

No Yes

If Yes, was the method appropriate? (See dictionary)

No Yes

RATE THIS SECTION	STRONG	MODERATE	WEAK
**See dictionary**	1	2	3

C) CONFOUNDERS

(Q1) Were there important differences between groups prior to the intervention?

1 Yes
2 No
3 Can't tell

The following are examples of confounders:

1 Race
2 Sex
3 Marital status/family
4 Age
5 SES (income or class)
6 Education
7 Health status
8 Pre-intervention score on outcome measure

(Q2) If yes, indicate the percentage of relevant confounders that were controlled (either in the design (e.g. stratification, matching) or analysis)?

1 80 – 100% (most)
2 60 – 79% (some)
3 Less than 60% (few or none)
4 Can't Tell

RATE THIS SECTION	STRONG	MODERATE	WEAK
**See dictionary**	1	2	3

D) BLINDING

(Q1) Was (were) the outcome assessor(s) aware of the intervention or exposure status of participants?

1 Yes
2 No
3 Can't tell

(Q2) Were the study participants aware of the research question?

1 Yes
2 No
3 Can't tell

RATE THIS SECTION	STRONG	MODERATE	WEAK
**See dictionary**	1	2	3

E) DATA COLLECTION METHODS

(Q1) Were data collection tools shown to be valid?

1 Yes

2 No
3 Can't tell

(Q2) Were data collection tools shown to be reliable?
1 Yes
2 No
3 Can't tell

RATE THIS SECTION	STRONG	MODERATE	WEAK
**See dictionary**	1	2	3

F) WITHDRAWALS AND DROP-OUTS

(Q1) Were withdrawals and drop-outs reported in terms of numbers and/or reasons per group?
1 Yes
2 No
3 Can't tell
4 Not Applicable (i.e. one time surveys or interviews)

(Q2) Indicate the percentage of participants completing the study. (If the percentage differs by groups, record the lowest).
1 80 -100%
2 60 - 79%
3 less than 60%
4 Can't tell
5 Not Applicable (i.e. Retrospective case-control)

RATE THIS SECTION	STRONG	MODERATE	WEAK	
**See dictionary**	1	2	3	Not Applicable

G) INTERVENTION INTEGRITY

(Q1) What percentage of participants received the allocated intervention or exposure of interest?
1 80 -100%
2 60 - 79%
3 less than 60%
4 Can't tell

(Q2) Was the consistency of the intervention measured?
1 Yes
2 No
3 Can't tell

(Q3) Is it likely that subjects received an unintended intervention (contamination or co-intervention) that may influence the results?
4 Yes
5 No
6 Can't tell

H) ANALYSES

(Q1) Indicate the unit of allocation (circle one)
community organization/institution practice/office individu

(Q2) Indicate the unit of analysis (circle one)
community organization/institution practice/office individu

(Q3) Are the statistical methods appropriate for the study design?
1 Yes
2 No
3 Can't tell

(Q4) Is the analysis performed by intervention allocation status (i.e. intention to treat) rather than the actual intervention received?
1 Yes
2 No
3 Can't tell

GLOBAL RATING

COMPONENT RATINGS
Please transcribe the information from the gray boxes on pages 1-4 onto this page. See dictionary on how to rate this section.

**A**	**SELECTION BIAS**	**STRONG**	**MODERATE**	**WEAK**	
		1	2	3	
**B**	**STUDY DESIGN**	**STRONG**	**MODERATE**	**WEAK**	
		1	2	3	
**C**	**CONFOUNDERS**	**STRONG**	**MODERATE**	**WEAK**	
		1	2	3	
**D**	**BLINDING**	**STRONG**	**MODERATE**	**WEAK**	
		1	2	3	
**E**	**DATA COLLECTION METHOD**	**STRONG**	**MODERATE**	**WEAK**	
		1	2	3	
**F**	**WITHDRAWALS AND DROPOUTS**	**STRONG**	**MODERATE**	**WEAK**	
		1	2	3	Not Applicable

<u>GLOBAL RATING FOR THIS PAPER (circle one):</u>

1	STRONG	(no WEAK ratings)
2	MODERATE	(one WEAK rating)
3	WEAK	(two or more WEAK ratings)

With both reviewers discussing the ratings:

Is there a discrepancy between the two reviewers with respect to the component (A-F) ratings?

No Yes

If yes, indicate the reason for the discrepancy

1 Oversight
2 Differences in interpretation of criteria
3 Differences in interpretation of study

Final decision of both reviewers (circle one):

1 **STRONG**
2 **MODERATE**
3 **WEAK**

D – EMBASE search string for DON

#25 #8 NOT [animals]/lim **298**

#24 #23 NOT #10 NOT #18 **31**

#23 #22 AND [2016-2020]/py **443**

#22 #21 AND english:la **1,052**

#21 #11 AND #20 **1,085**

#20 #19 OR #13 **22,871,744**

#19 [humans]/lim **21,993,373**

#18 #17 NOT #10 52

#17 #16 AND [2016-2020]/py 290

#16 #15 AND **english**:la 741

#15 #11 AND #14 760

#14 #12 OR #13 4,432,012

#13 '**human**':ti,ab OR '**urin***':ti,ab OR '**cohort**':ti,ab 4,432,012

#12 '**human[lim]**' 43

#11 '**vomitoxin**'/exp OR '**vomitoxin***':ti OR '**deoxynivalenol**'/exp/mj OR '**deoxynivalenol***':ti 3,472

#10 #8 NOT #9 378

#9 '**in vitro**' 2,196,997

#8 #7 AND [2016-2020]/py 465

#7 #6 AND **english**:la 1,163

#6 #1 AND #5 1,196

#5 #2 OR #4 23,170,518

#4 '**human**':ti,ab OR '**urin***':ti,ab OR '**cohort**':ti,ab 4,432,012

#3 #1 AND #2 1,146

#2 '**human**' 22,962,843

#1 '**vomitoxin**'/exp OR **vomitoxin** OR '**deoxynivalenol**'/exp/mj OR '**deoxynivalenol**':ti 3,494

E – EMBASE search string for FB1

Embase search

#**5** #3 NOT #4 729

#**4** '**arabidopsis**':ti,ab 60,878

#**3** #1 AND #2 752

#**2** '**fumonisin**' 3,858

#**1** '**sphingolipid**' OR '**sphingolipid metabolism**' OR '**sphingosine**' OR '**sphinganine**' 28,019

Embase search for other ceramide synthase inhibitors

#3 #1 AND #2 88

#2 'ceramide synthase' OR 'sphingosine n-acyltransferase' OR 'sphinganine n-acyltransferase' 1,013

#1 'fty720'/exp OR 'fty720' OR 'fingolimod'/exp OR 'fingolimod' OR 'gilenya'/exp
OR 'gilenya' OR 'myriocin'/exp OR 'myriocin' OR 'australifuncin*' OR 'aal toxin*' 10,979

Embase search for AAL

#**3** #1 AND #2 18

#**2** '**aal toxin**' 50

#**1** '**sphingosine**' OR '**spinganine**' OR '**ceramide synthase**' OR '**sphingolipid metabolism**' 19,279
